# Cerebrospinal Fluid Chitinases as Biomarkers for Amyotrophic Lateral Sclerosis

**DOI:** 10.3390/diagnostics11071210

**Published:** 2021-07-05

**Authors:** Júlia Costa, Marta Gromicho, Ana Pronto-Laborinho, Conceição Almeida, Ricardo A. Gomes, Ana C. L. Guerreiro, Abel Oliva, Susana Pinto, Mamede de Carvalho

**Affiliations:** 1 Instituto de Tecnologia Química e Biológica António Xavier, Universidade Nova de Lisboa, Avenida da República, 2780-157 Oeiras, Portugal; oliva@itqb.unl.pt; 2 Instituto de Fisiologia, Faculdade de Medicina, Universidade de Lisboa, 1649-028 Lisbon, Portugal; mgromichosilva@medicina.ulisboa.pt (M.G.); anapronto@medicina.ulisboa.pt (A.P.-L.); scandeias@medicina.ulisboa.pt (S.P.); mamedealves@medicina.ulisboa.pt (M.d.C.); 3 Instituto de Medicina Molecular, Faculdade de Medicina, Universidade de Lisboa, 1649-028 Lisbon, Portugal; 4 UniMS—Mass Spectrometry Unit, iBET—Instituto de Biologia Experimental e Tecnologica, 2780-157 Oeiras, Portugal; salmeida@itqb.unl.pt (C.A.); ragomes@ibet.pt (R.A.G.); aguerreiro@ibet.pt (A.C.L.G.); 5 UniMS—Mass Spectrometry Unit, ITQB—Instituto de Tecnologia Quimica e Biologica Antonio Xavier, Universidade Nova de Lisboa, 2780-157 Oeiras, Portugal; 6 Department of Neurosciences and Mental Health, Hospital de Santa Maria-CHULN, 1649-035 Lisbon, Portugal

**Keywords:** amyotrophic lateral sclerosis, chitinases, cerebrospinal fluid, biomarkers

## Abstract

Amyotrophic lateral sclerosis (ALS) is a neurodegenerative neuromuscular disease that affects motor neurons controlling voluntary muscles. Survival is usually 2–5 years after onset, and death occurs due to respiratory failure. The identification of biomarkers would be very useful to help in disease diagnosis and for patient stratification based on, e.g., progression rate, with implications in therapeutic trials. Neurofilaments constitute already-promising markers for ALS and, recently, chitinases have emerged as novel marker targets for the disease. Here, we investigated cerebrospinal fluid (CSF) chitinases as potential markers for ALS. Chitotriosidase (CHIT1), chitinase-3-like protein 1 (CHI3L1), chitinase-3-like protein 2 (CHI3L2) and the benchmark marker phosphoneurofilament heavy chain (pNFH) were quantified by an enzyme-linked immunosorbent assay (ELISA) from the CSF of 34 ALS patients and 24 control patients with other neurological diseases. CSF was also analyzed by UHPLC-mass spectrometry. All three chitinases, as well as pNFH, were found to correlate with disease progression rate. Furthermore, CHIT1 was elevated in ALS patients with high diagnostic performance, as was pNFH. On the other hand, CHIT1 correlated with forced vital capacity (FVC). The three chitinases correlated with pNFH, indicating a relation between degeneration and neuroinflammation. In conclusion, our results supported the value of CHIT1 as a diagnostic and progression rate biomarker, and its potential as respiratory function marker. The results opened novel perspectives to explore chitinases as biomarkers and their functional relevance in ALS.

## 1. Introduction

Amyotrophic lateral sclerosis (ALS) is a neurodegenerative disease that affects upper and lower motor neurons in the motor cortex, brainstem and spinal cord. Typical age of onset is between 50 and 70 years of age. It is an infrequent disease, with an incidence of 2–3/100,000; however, the lifetime risk is estimated at 1/350 in Europe [1]. Therapies include ventilatory and nutritional support. Riluzole and edaravone are the only licensed drugs, with only modest effects on survival and rate of progression, respectively. ALS is incurable and death generally occurs within 2–5 years after onset due to respiratory insufficiency and complications.

About 5–10% of ALS cases have a positive family history and more than 30 ALS genes have been associated with the disease [2]. The GGGGCC hexanucleotide repeat upstream of the C9ORF72 coding region is the most common cause of familial ALS and frontotemporal dementia. Other well-studied mutated genes are SOD1, TARDBP and FUS. Very recently, mutations in EGF domain-specific O-linked N-acetylglucosamine transferase [3] and glycosyltransferase 8 domain-containing 1 [4] have also been found in ALS. The remaining 90–95% of ALS cases are sporadic, for which mutations are also found in some patients.

Pathological mechanisms in ALS include dysregulation of DNA and RNA metabolism, protein misfolding and aggregation, endoplasmic reticulum stress, proteasome inhibition and autophagy, neurofilament accumulation and impaired axonal transport, mitochondria damage and apoptosis, oxidative stress, excitotoxicity and neuroinflammation [5,6]. Death of the motor neuron is non-cell autonomous and also depends on surrounding glia and possibly other cell types [7,8]. Neuroinflammation plays an important role in familial and sporadic ALS pathogenesis, as in other neurodegenerative diseases. Neuroinflammation in ALS is mostly characterized by the activation of microglia and astroglia, but peripheral immune cells infiltrating the CNS, including lymphocytes, macrophages and natural killer cells, also play an important role. Microglia that, in healthy conditions, play a supportive role to maintain neuron homeostasis, with disease initiation due to different factors (e.g., ALS-associated mutations), acquire toxic properties, inducing neuron damage. Concomitantly, deregulation of secretory factors occurs, such as imbalanced pro- versus anti-inflammatory cytokine profiles and growth factors [8,9,10,11].

Although mammals do not contain endogenous chitin or chitin synthases genes, several cells in the human body, including activated immune cells, are capable of producing chitinases [12]. Chitinases in mammals belong to the glycoside hydrolase 18 family (CAZy, GH18). They include two true chitinases (chitotriosidase, CHIT1, and acidic mammalian chitinase, AMCase) that are catalytically active, as well as chitin-like proteins or chitolectins that still bind chitin but do not present hydrolytic activity. CHIT1 (EC 3.2.1.14) cleaves glycosidic linkages in chitin, which is a linear polymer of β1,4 linked N-acetylglucosamine (GlcNAc). CHIT1 was detected in microglia from the corticospinal tract [13] in macrophages and neutrophils [12]. Chitin-like proteins include CHI3L1 (YKL-40) and CHI3L2 (YKL-39) [14]. CHI3L1 was found to bind heparin and play a role in cell signaling [15]. CHI3L1 was detected in a subset of activated astrocytes in the white matter of the motor cortex and spinal cord of ALS patients [16], as well as in monocytes/macrophages, chondrocytes, synovial cells and osteoclasts [12]. CHI3L2 has been detected in macrophages, tumor cells [17] and cartilage chondrocytes [12].

The search for ALS biomarkers has been a field of intensive research, either by hypothesis-driven approaches related to disease pathology or by unbiased systematic analyses (omics). Currently, neurofilaments (NF), which are increased in CSF and blood of ALS patients as consequence of motor neuron damage [18], are largely accepted as biomarkers for ALS; they constitute benchmark biomarkers and have been used in clinical trials [19]. This has been supported by numerous studies from different groups and multicenter studies validating the phosphoneurofilament heavy chain (pNFH) and neurofilament light chain (NFL) as ALS biomarkers [20,21,22,23,24,25]. Unfortunately, NF are also increased in other neurological diseases, driving the need to find additional targets. The value of chitinases as biomarkers for amyotrophic lateral sclerosis has been supported by strong recent evidence [12,26,27]. CHIT1 has been advanced as an ALS biomarker in diagnosis and progression [13,16,28,29,30,31], as has CHI3L1 [16,29,30]. Although less studied, CHI3L2 [29,30,32] also appeared promising. In this context, even if CSF CHIT1 is known to be active towards synthetic substrates in vitro [30], its potential substrates in vivo have not been identified yet. Thus, the functional role of chitinases in ALS is still unknown [8,27].

Here, we investigated CSF levels of chitinases CHIT1, CHI3L1 and CHI3L2, testing their biomarker potential for ALS. In addition, we analyzed the CSF of ALS patients by UHPLC-mass spectrometry to investigate potential products of CHIT1 activity.

## 2. Materials and Methods

### 2.1. Patient Material

Cerebrospinal fluid was collected by lumbar puncture into polypropylene tubes without additives and immediately stored at −80 °C. In all included subjects, serology for Borrelia burgdoferi and Treponema pallidum (CSF) and retrovirus (blood) were negative. We included the following groups of subjects: 37 ALS patients; 24 controls with other neurological diseases—polyneuropathy (15); sudden headache with normal diagnostic workup (3); normal pressure hydrocephalus (1); ganglionopathy (1); myelitis (1); primary progressive multiple sclerosis (MS) (1); mitochondriopathy (1); brachial plexopathy (1) (Table 1). All ALS patients included were regularly followed at the Neuromuscular Unit of our hospital. ALS patients presented probable or definite disease, according to the revised El Escorial criteria [33]. All patients had spinal onset, except for one who had bulbar onset. At the time of CSF sampling, the patients were observed and disease severity was scored by applying ALSFRS (Amyotrophic Lateral Sclerosis Functional Rating Scale), and forced vital capacity (FVC) was registered. CSF was collected as part of the diagnostic workup. Patients were above 18 years old and all signed a written informed consent before performing the lumbar puncture, which was performed as part of the diagnosis workup. Patients with other medical conditions, on gastrostomy, taking supplements other than vitamins or with symptoms of respiratory distress or cognitive changes were excluded.

### 2.2. ELISA Quantifications

For ELISA assays, CSF was centrifuged at 2000× *g* for 10 min, RT, and the supernatant used for quantification. pNFH from the CSF was quantified using the ELISA kit from BioVendor Research and Diagnostic Products (RD191138300R, Brno, Czech Republic), as previously described [21]. CHIT1, CHI3L1 and CHI3L2 were quantified using a CircuLex ELISA kit (MBL, CY-8074; Nagoya, Japan), Quantikine ELISA kit (R&D, DC3L10; Minneapolis, MN, USA) and CircuLex ELISA kit (MBL, CY-8087), respectively, following the supplier’s instructions. Calibration curves between 56.25 and 3600 pg/mL for CHIT1, between 62.5 and 4000 pg/mL for CHI3L1 and between 37.5 and 2400 pg/mL for CHI3L2 were performed. Measurements were conducted in duplicate and CV was typically below 10%. CHIT1, CHI3L1 and CHI3L2 concentrations were calculated from interpolation in four-parameter logistic (4PL) non-linear regression curves of log (concentration) versus (absorbance 450–absorbance 540) curve (GraphPad Prism 9) (r2 above 0.998, 0.999 and 0.992, respectively). CSF dilution was adjusted to meet this criterion; typical dilutions used were 1:15, 1:300 and 1:25 for CHIT1, CHI3L1 and CHI3L2, respectively. For 2 controls and 2 patients, CHIT1 values were below the lower concentration of the calibration curve and were considered as zero for the calculations. These patients could correspond to homozygous CHIT1 duplication mutation carriers [28].

### 2.3. Statistical Analysis

Normality was checked by the D’Agostino and Pearson omnibus normality test and some sample distributions were not normal. Biomarker concentration was presented as median and the interquartile range (IQR, 25–75% percentiles). Progression rate was calculated as follows (40—ALSFRS)/disease duration). Statistical comparisons applied nonparametric Mann–Whitney test and Spearman’s non-parametric correlation analysis. Receptor operator characteristic (ROC) curve analysis was performed, and area under the curve was (AUC) calculated. Values of *p* < 0.05 were considered significant. Kaplan–Meier survival curves and the log-rank (Mantel–Cox) test were used to compare survival in patients with CSF levels of pNFH and each chitinase marker above and below medium value. Onset age and disease duration at sampling were entered as covariables in the Cox model. Statistical analysis was conducted with GraphPad Prism 9 (GraphPad Software, San Diego, CA, USA) and MedCalc software (Ostend, Belgium).

### 2.4. UHPLC-MS Analysis of CSF

Two CSF pools of 280 and 550 µL from distinct ALS patients tested in this work were used for oligosaccharide isolation. CHIT1 activities of the pools were 14911–97,420 and 28,195 pg/mL, respectively. CSF was centrifuged at 10,000× *g* for 10 min and applied onto reverse–phase C18 cartridges (50 mg; Waters; Milford, MA, USA) pre-conditioned with 60% acetonitrile (ACN), 0.5% trifluoroacetic acid (TFA), followed by 60% ACN, 0.5% TFA (two-fold 0.75 mL each) and water (three-fold 1 mL). Flow-through was re-applied once. Cartridges were washed with 0.1% TFA (two-fold 0.5 mL). The flow-through and the two washes, which contained the oligosaccharides, were pooled, neutralized with 2.5% ammonia and dried in the Speed-Vac concentrator. This fraction was solubilized in 0.8 mL water and applied onto Hypercarb cartridges (25 mg; ThermoFisher Scientific; Waltham, MA USA) pre-conditioned with 0.8 mL ACN and 0.1% TFA and water (three-fold 0.8 mL), as previously described [34]. The cartridges were washed with water (three-fold 0.8 mL) and the bound oligosaccharides were eluted with 0.8 mL 25% can, followed by 0.8 mL 40% ACN, 0.1% TFA and finally 0.8 mL 80% can and 0.1% TFA. The Hypercarb cartridges bound disaccharides and larger oligosaccharides but not monosaccharides. The eluates were neutralized with 2.5% ammonia and dried in the Speed-Vac concentrator.

Chromatographic analysis of CSF oligosaccharides was performed on an UltiMate 3000 UHPLC (Thermo Scientific, ThermoFisher Scientific; Waltham, MA, USA). The separation was performed using a Thermo column Hypercarb (2.1 × 100 mm, 3 µm particle size, P/N 36003-102130). The mobile phase A was water with 0.1% formic acid (*v*/*v*), and mobile phase B was acetonitrile with 0.1% formic acid (*v*/*v*) (Optima™ LC/MS Grade, Fisher Scientific, ThermoFisher Scientific; Waltham, MA USA). The gradient was as follows: 0 to 1 min 99% A and 1% B; 1 to 13 min decrease to 1% A, which was maintained until 15 min; 15 to 16 min gradient to 99% A, which was maintained until 20 min. The column temperature was 30 °C, and a flow rate of 400 µL/min was used.

The data were acquired on a Q Exactive Focus (Thermo Scientific) coupled to UHPLC, using Xcalibur software v.4.0.27.19 (Thermo Scientific). The method consisted the Full MS scan (R = 70,000) and ddMS2 (data-dependent MS2) in negative mode. External calibration was performed using LTQ ESI Negative Ion Calibration Solution (Thermo Scientific) and the lock mass enabled internal calibration. The raw HRAM (high-resolution accurate-mass) data were analyzed using Compound Discoverer software v3.2 (Thermo Scientific). The data analysis workflow employed the mass list and mzVault nodes populated with databases containing the compounds of interest. The compound database search was performed with a 5 ppm mass tolerance. The mark background node was also employed to filter out background compounds identified in the blank samples.

Hypercarb bound fractions were screened for the presence of: Galβ1-4GlcNAc (LacNAc) and GalNAcβ1-4GlcNAc (LacdiNAc) potentially resulting from endogenous glycolipids or glycoproteins [35]; chitooligosaccharides (di- to hexasaccharides) that would result from the hydrolysis of chitin, which is a homopolymer of GlcNAcβ1-4GlcNAc [36]; di- to hexasaccharides that would result from the hydrolysis of hyaluronan, which is a polymer of disaccharide repeating units GlcNAcβ1-4GlcAβ1-3. As external standard to validate the analysis, we used monosaccharide N-glycolylneuraminic acid, which is absent from human samples [34].

## 3. Results

### 3.1. Chitinases and pNFH Analysis from the CSF

Chitinases and pNFH were quantified by ELISA in ALS patients and in disease control patients. Demographic data are presented in Table 1. Age was similar between groups (*p* > 0.05).

**Figure 1:**
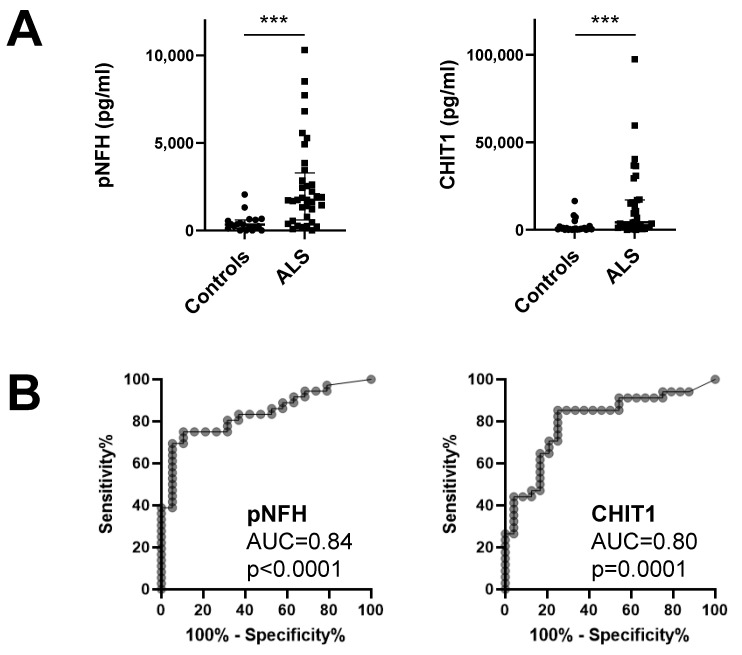
Levels of pNFH and CHIT1 from the CSF of ALS patients: (**A**) Comparison between patients with ALS and controls with other diseases. (**B**) ROC curve analysis of pNFH and CHIT1. AUC represents area under the curve. ***, *p* ≤ 0.001.

The median level of CHIT1 was 6.7-fold higher in ALS patients (4254 pg/mL) than in disease controls (638.9 pg/mL) (*p* < 0.0001) (Table 1; Figure 1A). ROC curve analysis showed that CHIT1 diagnostic performance was good (AUC 0.80, *p* = 0.0001) (Figure 1B). CHI3L1 and CHI3L2 levels were also higher in the ALS group but the difference did not reach statistical significance. Concerning the benchmark marker, pNFH, the median level was 5.2-fold higher in ALS patients (1751 pg/mL) than in controls (338.7 pg/mL), and its diagnostic performance was good (AUC = 0.84, *p* < 0.0001) (Figure 1A,B).

One control patient with multiple sclerosis displayed the highest level of CHIT1 (16,456 pg/mL), CHI3L1 (357 ng/mL) and CHI3L2 (36 ng/mL), but not pNFH (355 pg/mL) (Figure 1A).

**Figure 2:**
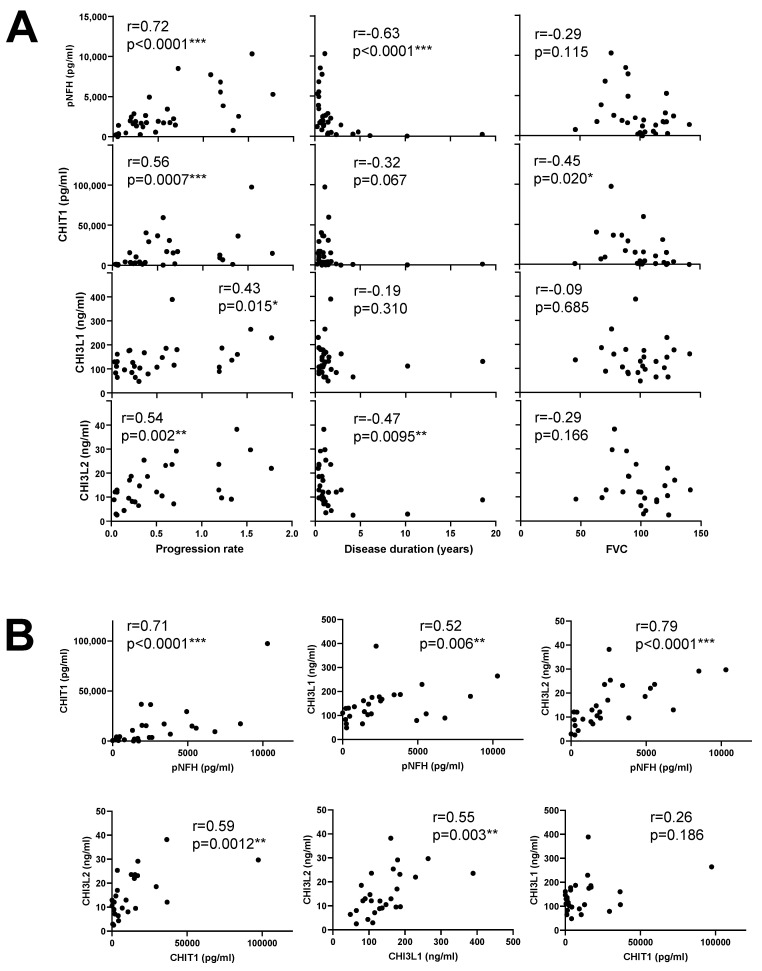
Correlation of pNFH, CHIT1, CHI3L1 and CHI3L2 from the CSF of ALS patients with clinical parameters (**A**) and with each other (**B**). Spearman’s r is presented. In (**A**), all values were considered for the calculations, whereas in (**B**), only values from ALS patients for which pNFH, CHIT1, CHI3L1 and CHI3L2 were all concomitantly available (*n* = 27) were considered. *, *p* ≤ 0.05; **, *p* ≤ 0.01; ***, *p* ≤ 0.001.

CHIT1 had a reasonable correlation with progression rate (r = 0.56, *p* = 0.0007), as did CHI3L2 (r = 0.54, *p* = 0.002) and CHI3L1 (r = 0.43, *p* = 0.015) (Figure 2A). As expected, pNFH had a high correlation with disease progression rate (r = 0.72, *p* < 0.0001). Furthermore, CHIT1 and CHI3L1 showed moderate but significant correlations with ALSFRS at baseline (r = −0.37, *p* = 0.036 and r = −0.38, *p* = 0.034, respectively). Concerning limb onset, no significant difference was found among left/right/lower/upper limbs. Among all markers, CHI3L2 presented a significant correlation with disease duration (r = −0.47, *p* = 0.0095), as did pNFH (r = −0.63, *p* < 0.0001) (Figure 2A).

Interestingly, we found a negative correlation between FVC and CHIT1 (r = −0.45, *p* = 0.020), which was not observed for other markers (Figure 2A). This is in line with a negative correlation between FVC and progression rate (r = −0.47, *p* = 0.008).

There was a high correlation between CHI3L2 and pNFH (r = 0.79, *p* < 0.0001), as well as for CHIT1 (r = 0.71, *p* < 0.0001). CHI3L1 had a reasonable correlation with pNFH (r = 0.52, *p* = 0.006). On the other hand, CHI3L2 correlated with CHIT1 (r = 0.59, *p* = 0.0012) and CHI3L1 (r = 0.55, *p* = 0.003) (Figure 2B), in contrast to CHIT1 versus CHI3L1.

**Figure 3:**
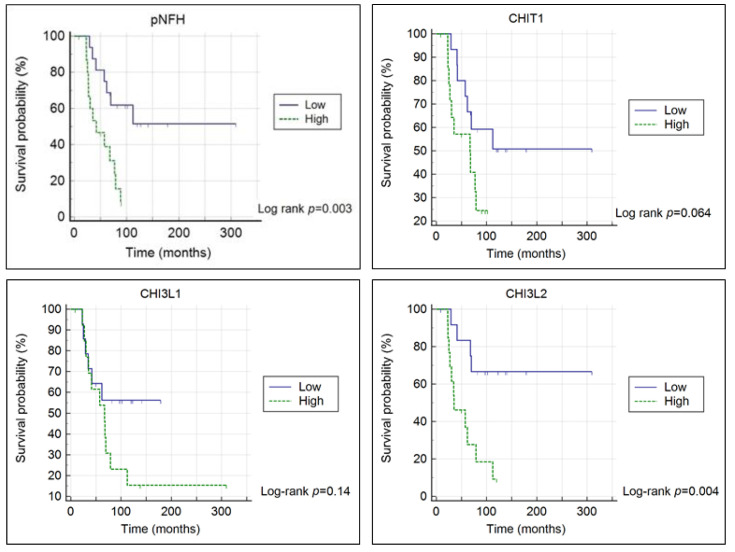
Kaplan–Meier survival curves stratified by low and high chitinase protein level (above or below or equal to the median level). Time in months since first symptoms were considered.

Survival of patients stratified by different levels of pNFH or CHI3L2 was significantly different (log-rank test: χ^2^ = 9.0999, *p* = 0.003; log-rank test: χ^2^ = 8.4278, *p* = 0.004, respectively), but not for CHIT1 or CHI3L1 (Figure 3).

Cox model showed that low CHI3L2 level was an independent predictor for survival, in addition to age and disease duration (CHI3L2; HR = 0.201, 95% CI = 0.0525 to 0.7705, *p* = 0.019) (Table 2).

### 3.2. UHPLC-MS Analysis of CSF

In an attempt to identify potential products of CHIT1 activity in the CSF, we fractionated CSF using reverse-phase C18 (to remove proteins and hydrophobic molecules) and graphite Hypercarb (that binds oligosaccharides) cartridges to obtain an oligosaccharide enriched fraction. Pools of CSF available from patients tested for CHIT1 were used. Hypercarb-bound fractions were analyzed by UHPLC-MS using a Hypercarb column. We screened for putative CHIT1 products, which included chito-oligosaccharides as hydrolysis products of chitin (linear polymer of β1-4 linked GlcNAc) [36], the disaccharides Galβ1-4GlcNAc and GalNAcβ1-4GlcNAc (LacdiNAc) as potential products of mamalian glycoproteins or glycolipids [35] and oligosaccharides resulting from the hydrolysis of hyaluronan (linear polymer of the repeating disaccharide GlcNAcβ1-4GlcAβ1-3) that is present in the CSF [37] and has some structural resemblance to chitin. From these, only a signal at *m*/*z* 469.166 [M+FA-H] compatible with HexNAc-HexNAc was detected (Supplementary Figure S1). This could correspond to di-N-acetylchitobiose (GlcNAcβ1-4GlcNAc) or LacdiNAc (GalNAcβ1-4GlcNAc). It was not possible to perform fragmentation to confirm the structure due to the low intensity of the signal.

## 4. Discussion

In this study, we presented evidence supporting the relevance of chitinases as ALS biomarkers. This comparative study highlighted the importance of different chitinases as potentially useful markers for distinct disease characteristics.

CHIT1 levels were elevated in ALS compared to disease controls, in agreement with reports by other groups [13,16,28,29,30,38]. Furthermore, a high diagnostic performance of CHIT1 was detected but it did not outperform pNFH. Most interestingly, we disclosed a significant negative correlation between CHIT1 and the respiratory function index of forced vital capacity (FVC). FVC measures potential respiratory impairment in ALS. Evidence from the literature has indicated that FVC value may constitute a predictor of survival and disease progression [39], which is further corroborated by our findings here. In this context, it will be necessary to validate these results in a larger and independent cohort of patients. For CHI3L1, there was not a significant increase, which is in agreement with others [16].

For CHI3L2, increased levels have been reported in the literature [30,32] but, in our study, although an increase was observed, it was not significant. On the other hand, we found CHI3L2 to be a significant and independent predictor of survival using Kaplan–Meier estimator and Cox proportional hazards modelling, which contrasted to CHIT1 and CHI3L1. For CHIT1, existing reports favor a predictive value of survival [30,38]. Concerning CHI3L1, there is discrepancy of results in the literature [29,30,38]. The significant relevance of CHI3L2 to predict survival in ALS observed in our patients differed from results of another group [29,30]. Since the number of independent studies investigating CHI3L2 is low in the literature, our results emphasize the need to further investigate this chitinase and its biomarker potential in larger independent cohorts of patients.

Although concentration values for all proteins were within the range described elsewhere [13,30], there were some differences between our study and others concerning the potential of different chitinases as biomakers for ALS. This was probably due to the number and subject characteristics of the control group with other diseases, since some of the controls may also exhibit chitinases induction. For example, in multiple sclerosis, an increase in CHIT1 has been reported in the literature [40], which is in agreement with our findings. Therefore, besides testing larger independent cohorts of ALS and control patients, it would also be relevant to analyze additional control groups (e.g., healthy controls).

The three tested chitinases correlated with disease progression rate in ALS patients, and CHIT1 and CHI3L2 showed the strongest correlations. These results were in agreement with other authors [13,16,29,30,38]. Therefore, these molecules constitute potentially useful biomarkers for progression rate, which is particularly useful in the context of precision medicine that has patient stratification for differentiated therapeutic trials in mind.

We found that all chitinases correlated with pNFH, with the correlation being highest for CHIT1 and CHI3L2. Since pNFH is a marker of neuronal damage [41], most importantly, these results supported a connection between neuronal damage and neuroinflammation. In ALS, neuroinflammation is characterized predominantly by the activation of microglia and astrocytes, and by the presence in the CNS of non-resident immune cells, including T cells, monocyte-derived macrophages and natural killer cells [11]. Many studies have shown a deregulation in the levels of cytokines in the CSF and blood of ALS patients, including interleukins, tumor necrosis factors and interferon gamma produced by different immune cells [10]. Glial cells that are normally supportive of neuron homeostasis may become neurotoxic during the initial stages of the disease; this process may be triggered, for example, by dysfunction and misfolding of mutant proteins associated with ALS (e.g., SOD1, TDP-43, dipeptide repeat proteins) and neuron damage. Activated glial cells and peripheral immune cells release toxic molecules, including pro-inflammatory cytokines (e.g., IL-1β), that affect motor neuron integrity [10]. Therefore, a complex interplay between neuronal damage and immune cell activation occurs in ALS.

Chitinases are secreted by activated glial cells, as well as by other cells of the immune system [12,13,16]. CHIT1 was detected in microglia from the corticospinal tract [13] and in macrophages [12]. CHI3L1 was found in a subset of activated astrocytes [16], monocytes/macrophages [12]. CHI3L2 has been detected in macrophages [17]; curiously, it has been reported in neuronal cells from the cerebral cortex (https://www.proteinatlas.org/ENSG00000064886-CHI3L2/tissue/cerebral+cortex#img), but its expression in ALS tissues has not been described so far. As such, the origin of CSF CHI3L2 is currently unknown. Possible interplay between pro-inflammatory cytokines and chitinases has been discussed [12], with implications for their functional activity. In this context, chitinases recently appeared as promising therapeutic targets. For example, inhibition of CHI3L1 with the compound K284-6111 prevented amyloid beta-induced neuroinflammation and impairment of recognition memory via inhibition of the NF-κB pathway in an Alzheimer’s disease mouse model [42]. In line with this, CHI3L1 was found to be neurotoxic towards cortical neurons but not immune cells [43]. By contrast, human CHIT1 and CHI3L1 promoted oligodendrogenesis from neural stem cells [44]. Additionally, CHIT1 had a protective role in an Alzheimer’s disease rat model and N9 microglia cells [45]. More studies need to be performed to evaluate whether chitinases are just bystanders resulting from immune cell activation, or whether they have a physiological relevant role.

CHIT1 has chitinolytic activity towards chitin and artificial substrates [36], as well as mammalian N-acetylglucosamine-containing glycoconjugates [35]. CHIT1 from human CSF is active in vitro towards a synthetic substrate [30] but its endogenous target(s) is not known. Our efforts aiming at identifying potential products of CHIT1 activity in the CSF of patients tested here indicated an *m*/*z* signal compatible with the disaccharide HexNAc-HexNAc, which could consist of di-N-acetylchitobiose (GlcNAcβ1-4GlcNAc, a hydrolysis product of chitin) or LacdiNAc (GalNAcβ1-4GlcNAc, found in some human glycoproteins), which are hydrolyzed by CHIT1 [35,36]. Humans do not produce chitin that is found in fungi; however, there has been evidence of fungal infection in the CNS of ALS patients [46], which could explain the origin of di-N-acetylchitobiose. On the other hand, evidence for chitin-like polysaccharides has been reported in Alzheimer’s disease [47]. However, it should also be considered that di-N-acetylchitobiose could originate from the degradation of other glycoconjugates, such as N-linked glycans from endogenous human glycoproteins. Since hyaluronan (polysaccharide of the repeating disaccharide of N-acetylglucosamine and D-glucuronic acid) has some structural resemblance with chitin and is present in the CSF [37], we also screened for potential hydrolysis products but found no signal compatible with those oligosaccharides. This was in agreement with in vitro data indicating that CHIT1 did not cleave hyaluronan [48]. Limitations of this preliminary study include the following: low volumes for testing; only a duplicate, but from two independent pools, was performed; CSF pools were analyzed instead of individual samples; only ALS samples and not controls for comparison were investigated. However, a methodology for the study of CSF-free glycans has been presented. Moreover, a novel perspective has been presented to further investigate whether the disaccharide HexNAc-HexNAc is found in independent ALS and control samples, as well as in the positive scenario, to unequivocally elucidate the corresponding structure by MS/MS and/or liquid chromatography techniques.

In conclusion, our results supported the importance of chitinases as biomarker targets in ALS. Particularly, CHIT1 was a promising biomarker for diagnosis, progression rate and respiratory function. These results provided novel perspectives to further explore the potential of chitinases as ALS biomarkers and their functional relevance, they and may have implications in other neurological diseases.

## Figures and Tables

**Table 1:** Demographic information and levels of pNFH and chitinases from the CSF of the patients included. Median and interquartile range are shown. Abbreviations are: C, controls; CC, concentration; M/F, male/female. Age is presented in years. ***, *p* ≤ 0.001.

	pNFH			CHIT1			CHI3L1			CHI3L2		
	M/F	Age	CC (pg/mL)	M/F	Age	CC (pg/mL)	M/F	Age	CC (ng/mL)	M/F	Age	CC (ng/mL)
C	11/8	62.3 (52.3–67.0)	338.7 (114.9–605.7)	12/12	55.9 (43.1–65.5)	638.9 (273.7–1678)	9/9	56.0 (45.8–67.0)	111.7 (84.3–147.7)	8/9	57.2 (47.7–67.0)	8.66 (6.47–12.13)
ALS	26/10	56.0 (48.0–66.4)	1751 (604.1–3285)	25/9	56.7 (47.4–66.3)	4254 (1293–17074)	24/8	58.0 (49.7–66.4)	128 (91.4–173.3)	22/8	59.4 (49.8–66.6)	12.10 (8.17–22.27)
*p*	-	0.420	<0.0001 ***	-	0.826	<0.0001 ***	-	0.909	0.515	-	0.991	0.099

**Table 2:** Cox proportional hazards modelling of factors known to be associated with survival and individual chitinase proteins separately. b—regression coefficient; SE—standard error, Wald statistic; HR—hazard ratio; CI—confidence interval.

Covariate	b	SE	Wald	*p*	HR	95% CI of HR
Age at onset	0.076	0.033	5.206	0.023	1.079	1.011 to 1.153
Disease duration at sampling	−0.099	0.043	5.428	0.020	0.906	0.833 to 0.984
CHIT1	−0.777	0.541	2.060	0.151	0.460	0.159 to 1.328
Age at onset	0.049	0.032	2.291	0.130	1.050	0.986 to 1.119
Disease duration at sampling	−0.096	0.042	5.301	0.021	0.908	0.837 to 0.986
CHI3L1	−0.398	0.531	0.562	0.453	0.672	0.237 to 1.902
Age at onset	0.115	0.055	4.454	0.035	1.122	1.008 to 1.249
Disease duration at sampling	−0.116	0.045	6.666	0.010	0.890	0.815 to 0.972
CHI3L2	−1.604	0.685	5.478	0.019	0.201	0.053 to 0.771
Age at onset	0.065	0.032	4.078	0.043	1.068	1.002 to 1.137
Disease duration at sampling	−0.059	0.033	3.264	0.071	0.942	0.884 to 1.005
pNFH	−0.890	0.537	2.741	0.098	0.411	0.143 to 1.178

## Data Availability

The data presented in this study are available in this article and supplementary material.

## Supplementary Materials

The following are available online at https://www.mdpi.com/article/10.3390/diagnostics11071210/s1, Figure S1: Full MS spectra with isotope pattern fit of HexNAc-HexNAc identified using Compound Discoverer 3.2 (Thermo).

## References

[1] Ryan M., Heverin M., McLaughlin R.L., Hardiman O. (2019). Lifetime Risk and Heritability of Amyotrophic Lateral Sclerosis. JAMA Neurol..

[2] Volk A.E., Weishaupt J.H., Andersen P.M., Ludolph A.C., Kubisch C. (2018). Current knowledge and recent insights into the genetic basis of amyotrophic lateral sclerosis. Med. Genet..

[3] Moll T., Shaw P.J., Cooper-Knock J. (2020). Disrupted glycosylation of lipids and proteins is a cause of neurodegeneration. Brain.

[4] Cooper-Knock J., Moll T., Ramesh T., Castelli L., Beer A., Robins H., Fox I., Niedermoser I., Van Damme P., Moisse M. (2019). Mutations in the Glycosyltransferase Domain of GLT8D1 Are Associated with Familial Amyotrophic Lateral Sclerosis. Cell Rep..

[5] Costa J., Gomes C., de Carvalho M. (2010). Diagnosis, pathogenesis and therapeutic targets in amyotrophic lateral sclerosis. CNS Neurol. Disord. Drug Targets.

[6] Taylor J.P., Brown R.H., Cleveland D.W. (2016). Decoding ALS: From genes to mechanism. Nature.

[7] Clement A.M., Nguyen M.D., Roberts E.A., Garcia M.L., Boillee S., Rule M., McMahon A.P., Doucette W., Siwek D., Ferrante R.J. (2003). Wild-type nonneuronal cells extend survival of SOD1 mutant motor neurons in ALS mice. Science.

[8] Vahsen B.F., Gray E., Thompson A.G., Ansorge O., Anthony D.C., Cowley S.A., Talbot K., Turner M.R. (2021). Non-neuronal cells in amyotrophic lateral sclerosis—From pathogenesis to biomarkers. Nat. Rev. Neurol..

[9] Beers D.R., Appel S.H. (2019). Immune dysregulation in amyotrophic lateral sclerosis: Mechanisms and emerging therapies. Lancet Neurol..

[10] Moreno-Garcia L., Miana-Mena F.J., Moreno-Martinez L., de la Torre M., Lunetta C., Tarlarini C., Zaragoza P., Calvo A.C., Osta R. (2021). Inflammasome in ALS Skeletal Muscle: NLRP3 as a Potential Biomarker. Int. J. Mol. Sci..

[11] McCauley M.E., Baloh R.H. (2019). Inflammation in ALS/FTD pathogenesis. Acta Neuropathol..

[12] Pinteac R., Montalban X., Comabella M. (2021). Chitinases and chitinase-like proteins as biomarkers in neurologic disorders. Neurol. Neuroimmunol. Neuroinflamm..

[13] Steinacker P., Verde F., Fang L., Feneberg E., Oeckl P., Roeber S., Anderl-Straub S., Danek A., Diehl-Schmid J., Fassbender K. (2018). Chitotriosidase (CHIT1) is increased in microglia and macrophages in spinal cord of amyotrophic lateral sclerosis and cerebrospinal fluid levels correlate with disease severity and progression. J. Neurol. Neurosurg. Psychiatry.

[14] Lee C.G., Da Silva C.A., Dela Cruz C.S., Ahangari F., Ma B., Kang M.J., He C.H., Takyar S., Elias J.A. (2011). Role of chitin and chitinase/chitinase-like proteins in inflammation, tissue remodeling, and injury. Annu. Rev. Physiol..

[15] Shao R., Hamel K., Petersen L., Cao Q.J., Arenas R.B., Bigelow C., Bentley B., Yan W. (2009). YKL-40, a secreted glycoprotein, promotes tumor angiogenesis. Oncogene.

[16] Vu L., An J., Kovalik T., Gendron T., Petrucelli L., Bowser R. (2020). Cross-sectional and longitudinal measures of chitinase proteins in amyotrophic lateral sclerosis and expression of CHI3L1 in activated astrocytes. J. Neurol. Neurosurg. Psychiatry.

[17] Liu L., Yang Y., Duan H., He J., Sun L., Hu W., Zeng J. (2021). CHI3L2 Is a Novel Prognostic Biomarker and Correlated With Immune Infiltrates in Gliomas. Front. Oncol..

[18] Gagliardi D., Meneri M., Saccomanno D., Bresolin N., Comi G.P., Corti S. (2019). Diagnostic and Prognostic Role of Blood and Cerebrospinal Fluid and Blood Neurofilaments in Amyotrophic Lateral Sclerosis: A Review of the Literature. Int. J. Mol. Sci..

[19] Chio A., Mazzini L., Mora G. (2020). Disease-modifying therapies in amyotrophic lateral sclerosis. Neuropharmacology.

[20] Feneberg E., Oeckl P., Steinacker P., Verde F., Barro C., Van Damme P., Gray E., Grosskreutz J., Jardel C., Kuhle J. (2018). Multicenter evaluation of neurofilaments in early symptom onset amyotrophic lateral sclerosis. Neurology.

[21] Goncalves M., Tillack L., de Carvalho M., Pinto S., Conradt H.S., Costa J. (2015). Phosphoneurofilament heavy chain and N-glycomics from the cerebrospinal fluid in amyotrophic lateral sclerosis. Clin. Chim. Acta.

[22] Goncalves M., De Carvalho M., Peixoto C., Alves P., Barreto C., Oliva A., Pinto S., Laborinho-Pronto A., Gromicho M., Costa J. (2017). Phosphoneurofilament heavy chain and vascular endothelial growth factor as cerebrospinal fluid biomarkers for ALS. Amyotroph. Lateral Scler. Frontotemporal. Degener..

[23] Lu C.H., Macdonald-Wallis C., Gray E., Pearce N., Petzold A., Norgren N., Giovannoni G., Fratta P., Sidle K., Fish M. (2015). Neurofilament light chain: A prognostic biomarker in amyotrophic lateral sclerosis. Neurology.

[24] Oeckl P., Jardel C., Salachas F., Lamari F., Andersen P.M., Bowser R., de Carvalho M., Costa J., van Damme P., Gray E. (2016). Multicenter validation of CSF neurofilaments as diagnostic biomarkers for ALS. Amyotroph. Lateral Scler. Frontotemporal. Degener..

[25] Steinacker P., Feneberg E., Weishaupt J., Brettschneider J., Tumani H., Andersen P.M., von Arnim C.A., Bohm S., Kassubek J., Kubisch C. (2016). Neurofilaments in the diagnosis of motoneuron diseases: A prospective study on 455 patients. J. Neurol. Neurosurg. Psychiatry.

[26] Gaur N., Perner C., Witte O.W., Grosskreutz J. (2020). The Chitinases as Biomarkers for Amyotrophic Lateral Sclerosis: Signals From the CNS and Beyond. Front. Neurol..

[27] Swash M. (2020). Chitinases, neuroinflammation and biomarkers in ALS. J. Neurol. Neurosurg. Psychiatry.

[28] Steinacker P., Feneberg E., Halbgebauer S., Witzel S., Verde F., Oeckl P., Van Damme P., Gaur N., Gray E., Grosskreutz J. (2021). Chitotriosidase as biomarker for early stage amyotrophic lateral sclerosis: A multicenter study. Amyotroph. Lateral Scler. Frontotemporal. Degener..

[29] Thompson A.G., Gray E., Thezenas M.L., Charles P.D., Evetts S., Hu M.T., Talbot K., Fischer R., Kessler B.M., Turner M.R. (2018). Cerebrospinal fluid macrophage biomarkers in amyotrophic lateral sclerosis. Ann. Neurol..

[30] Thompson A.G., Gray E., Bampton A., Raciborska D., Talbot K., Turner M.R. (2019). CSF chitinase proteins in amyotrophic lateral sclerosis. J. Neurol. Neurosurg. Psychiatry.

[31] Varghese A.M., Sharma A., Mishra P., Vijayalakshmi K., Harsha H.C., Sathyaprabha T.N., Bharath S.M., Nalini A., Alladi P.A., Raju T.R. (2013). Chitotriosidase—A putative biomarker for sporadic amyotrophic lateral sclerosis. Clin. Frontotemporal..

[32] Barschke P., Oeckl P., Steinacker P., Al Shweiki M.R., Weishaupt J.H., Landwehrmeyer G.B., Anderl-Straub S., Weydt P., Diehl-Schmid J., Danek A. (2020). Different CSF protein profiles in amyotrophic lateral sclerosis and frontotemporal dementia with C9orf72 hexanucleotide repeat expansion. J. Neurol. Neurosurg. Psychiatry.

[33] Brooks B.R., Miller R.G., Swash M., Munsat T.L., World Federation of Neurology Research Group on Motor Neuron Disease (2000). El Escorial revisited: Revised criteria for the diagnosis of amyotrophic lateral sclerosis. Amyotroph Lateral Scler Other Motor Neuron Disord.

[34] Costa J., Gatermann M., Nimtz M., Kandzia S., Glatzel M., Conradt H.S. (2018). N-Glycosylation of Extracellular Vesicles from HEK-293 and Glioma Cell Lines. Anal Chem.

[35] Larsen T., Yoshimura Y., Voldborg B.G., Cazzamali G., Bovin N.V., Westerlind U., Palcic M.M., Leisner J.J. (2014). Human chitotriosidase CHIT1 cross reacts with mammalian-like substrates. FEBS Lett.

[36] Renkema G.H., Boot R.G., Muijsers A.O., Donker-Koopman W.E., Aerts J.M. (1995). Purification and characterization of human chitotriosidase, a novel member of the chitinase family of proteins. J. Biol. Chem..

[37] Yu Y., Zhang F., Colon W., Linhardt R.J., Xia K. (2019). Glycosaminoglycans in human cerebrospinal fluid determined by LC-MS/MS MRM. Anal. Biochem..

[38] Gille B., De Schaepdryver M., Dedeene L., Goossens J., Claeys K.G., Van Den Bosch L., Tournoy J., Van Damme P., Poesen K. (2019). Inflammatory markers in cerebrospinal fluid: Independent prognostic biomarkers in amyotrophic lateral sclerosis?. J. Neurol. Neurosurg. Psychiatry.

[39] Czaplinski A., Yen A.A., Appel S.H. (2006). Forced vital capacity (FVC) as an indicator of survival and disease progression in an ALS clinic population. J. Neurol. Neurosurg. Psychiatry.

[40] Oldoni E., Smets I., Mallants K., Vandebergh M., Van Horebeek L., Poesen K., Dupont P., Dubois B., Goris A. (2020). CHIT1 at Diagnosis Reflects Long-Term Multiple Sclerosis Disease Activity. Ann. Neurol..

[41] Khalil M., Teunissen C.E., Otto M., Piehl F., Sormani M.P., Gattringer T., Barro C., Kappos L., Comabella M., Fazekas F. (2018). Neurofilaments as biomarkers in neurological disorders. Nat. Rev. Neurol..

[42] Choi J.Y., Yeo I.J., Kim K.C., Choi W.R., Jung J.K., Han S.B., Hong J.T. (2018). K284-6111 prevents the amyloid beta-induced neuroinflammation and impairment of recognition memory through inhibition of NF-kappaB-mediated CHI3L1 expression. J Neuroinflamm..

[43] Matute-Blanch C., Calvo-Barreiro L., Carballo-Carbajal I., Gonzalo R., Sanchez A., Vila M., Montalban X., Comabella M. (2020). Chitinase 3-like 1 is neurotoxic in primary cultured neurons. Sci. Rep..

[44] Starossom S.C., Campo Garcia J., Woelfle T., Romero-Suarez S., Olah M., Watanabe F., Cao L., Yeste A., Tukker J.J., Quintana F.J. (2019). Chi3l3 induces oligodendrogenesis in an experimental model of autoimmune neuroinflammation. Nat. Commun..

[45] Xiao Q., Yu W., Tian Q., Fu X., Wang X., Gu M., Lu Y. (2017). Chitinase1 contributed to a potential protection via microglia polarization and Abeta oligomer reduction in D-galactose and aluminum-induced rat model with cognitive impairments. Neuroscience.

[46] Alonso R., Pisa D., Fernandez-Fernandez A.M., Rabano A., Carrasco L. (2017). Fungal infection in neural tissue of patients with amyotrophic lateral sclerosis. Neurobiol. Dis..

[47] Castellani R.J., Perry G., Smith M.A. (2007). The role of novel chitin-like polysaccharides in Alzheimer disease. Neurotox. Res..

[48] Danielson B., Chen C.H., Kaber G., Mochly-Rosen D., Grimes K., Stern R., Bollyky P.L. (2018). Human Chitotriosidase Does Not Catabolize Hyaluronan. Int. J. Biol. Macromol..

