# Supplementary material for: Cerebrospinal Fluid Chitinases as Biomarkers for Amyotrophic Lateral Sclerosis
Source: Diagnostics (Basel). 2021 Jul 5;11(7):1210. doi: 10.3390/diagnostics11071210 (PMC8305219; doi:10.3390/diagnostics11071210)
Supplement: Supplementary file 1 [file diagnostics-11-01210-s001.zip › Figure S1_250521.pdf]

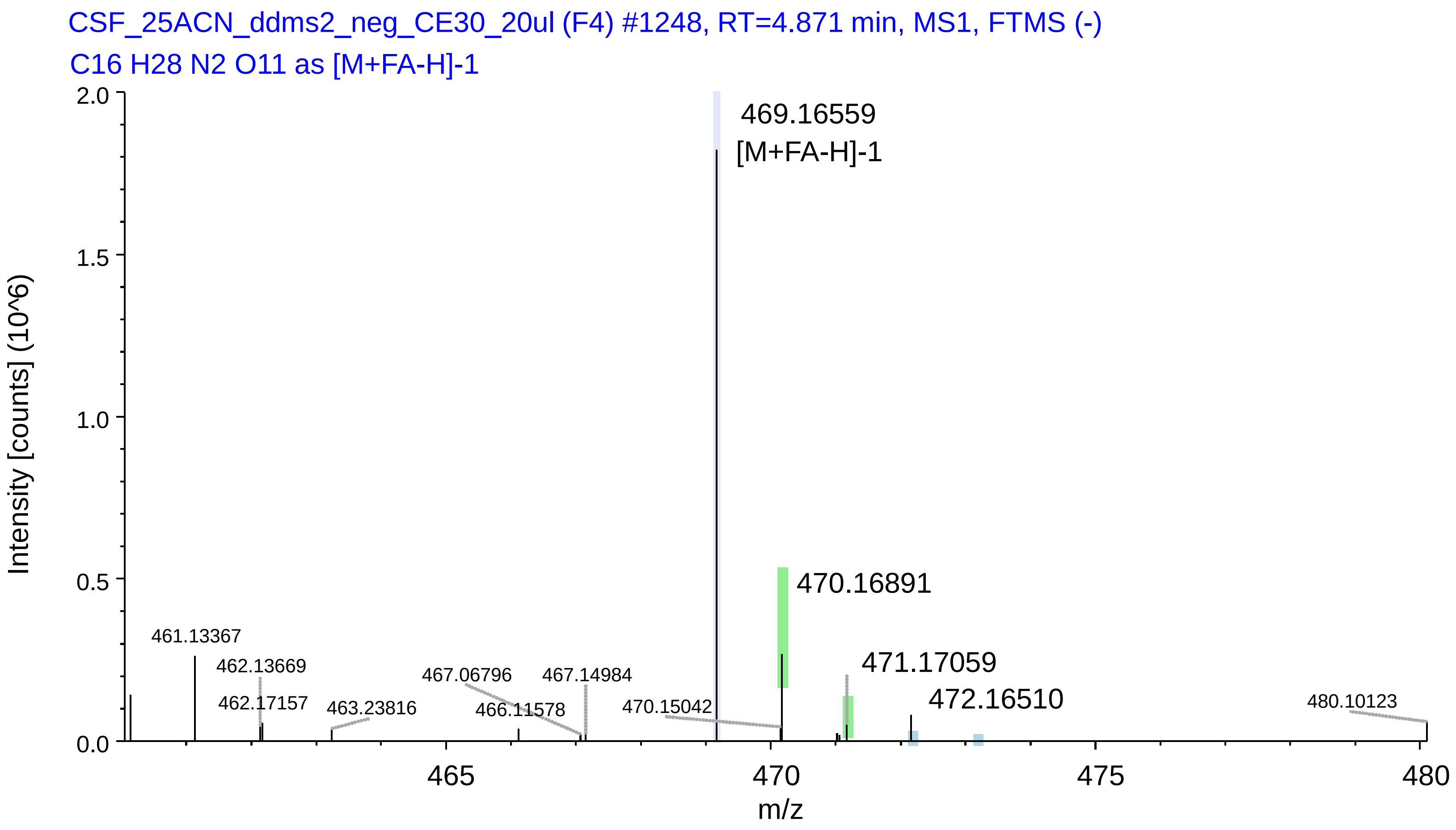

Figure S1: Full MS spectra with isotope pattern fit of HexNAc-HexNAc identified using Compound Discoverer 3.2 (Thermo). The green shade in the isotopic pattern represents a match between the predicted and the observed *m/z*. HexNAc, N-acetylhexosamine.
